# Seymour Fracture in a Pediatric Patient: A Case Report

**DOI:** 10.7759/cureus.10687

**Published:** 2020-09-27

**Authors:** Sumedha Bandi, Emily Drone, Ariel Vera, Latha Ganti

**Affiliations:** 1 Emergency Medicine, University of Minnesota, Minneapolis, USA; 2 Emergency Medicine, University of Central Florida/HCA Healthcare Osceola Regional Medical Center, Orlando, USA; 3 Emergency Medicine, Envision Physician Services, Nashville, USA; 4 Emergency Medical Services, Polk County Fire Rescue, Bartow, USA

**Keywords:** seymour fracture, mallet injury

## Abstract

In this report, the authors present the case of a child who sustained a specific type of mallet finger injury known as a Seymour fracture. This is an important injury to recognize in the emergency department as it is associated with significant morbidity if not treated appropriately. This is especially of concern in children, where the tissue can get trapped in the growth plate. Children also face a high risk of deformity due to growth arrest. Management includes thorough washout, reduction of displacement, and antibiotics and tetanus prophylaxis if there is an open fracture.

## Introduction

Pediatric hand fractures make up 2.3% of all ED visits [1]; these are more common in boys [2], with the most common mechanism being those secondary to sports-related activities. Phalangeal fractures are most common in the 9-12-year age group, and the most commonly affected finger is the middle finger [1].

A mallet finger injury is one of the most common injuries and is frequently associated with sport-related activities. One specific type of mallet finger injury, the Seymour fracture, results from a juxta epiphyseal fracture on the distal phalanx of the finger [1]. This injury involves avulsion of the nail and is usually classified as a Salter-Harris grade I or II fracture.

Early evaluation and appropriate treatment of a Seymour fracture include the administration of tetanus and antibiotic prophylaxis, and prompt orthopedic consultation [3]. Seymour fractures can reliably be identified with a good lateral X-ray and a high degree of suspicion.

## Case presentation

**Figure 1:**
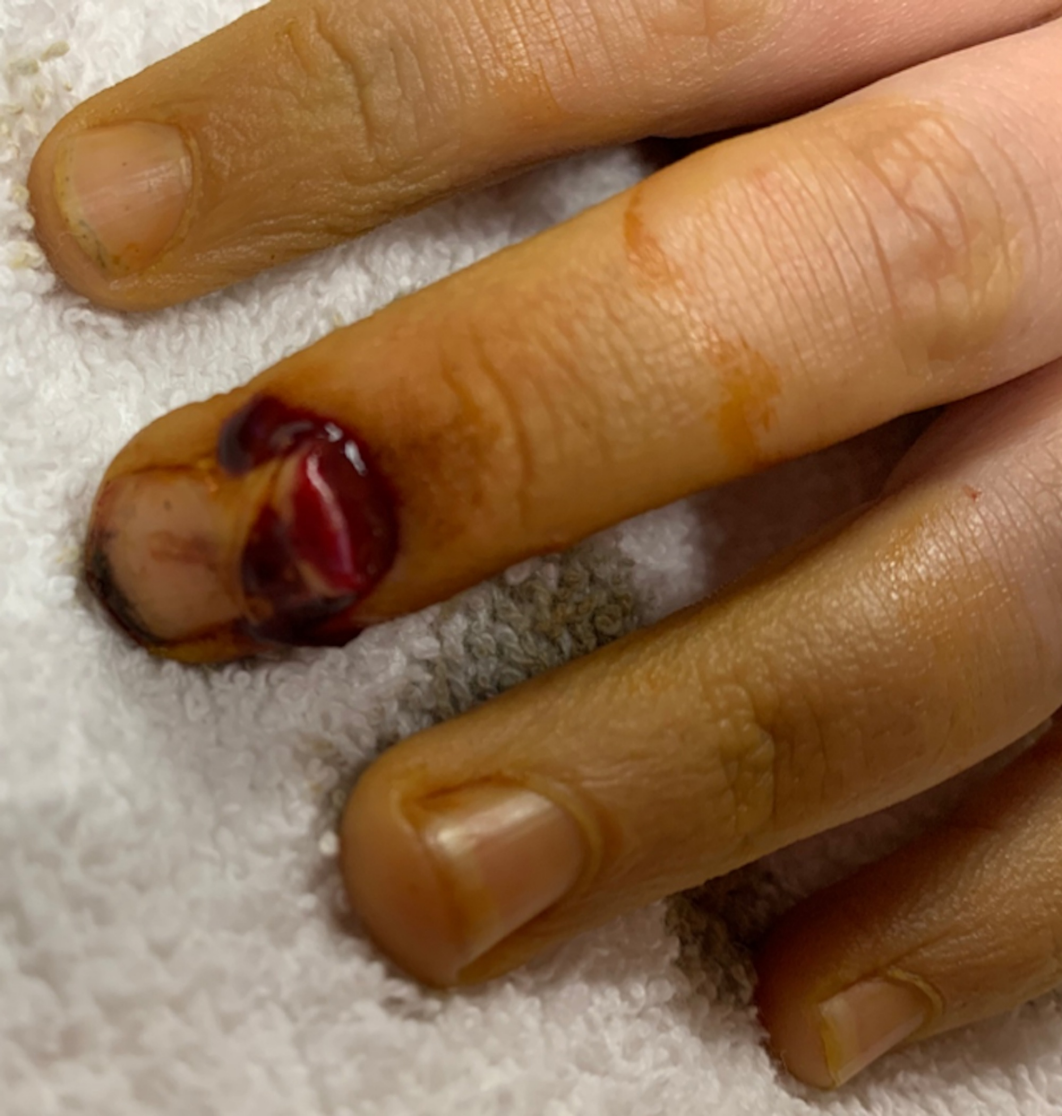
Photograph showing avulsion of the distal phalanx through the skin

A school-aged boy presented to the emergency department after catching his middle finger of his left hand in a hoverboard, which had caused avulsion of the distal phalanx through the skin. The bone was seen protruding through the bleeding, injured nailbed (Figure 1).

**Figure 2:**
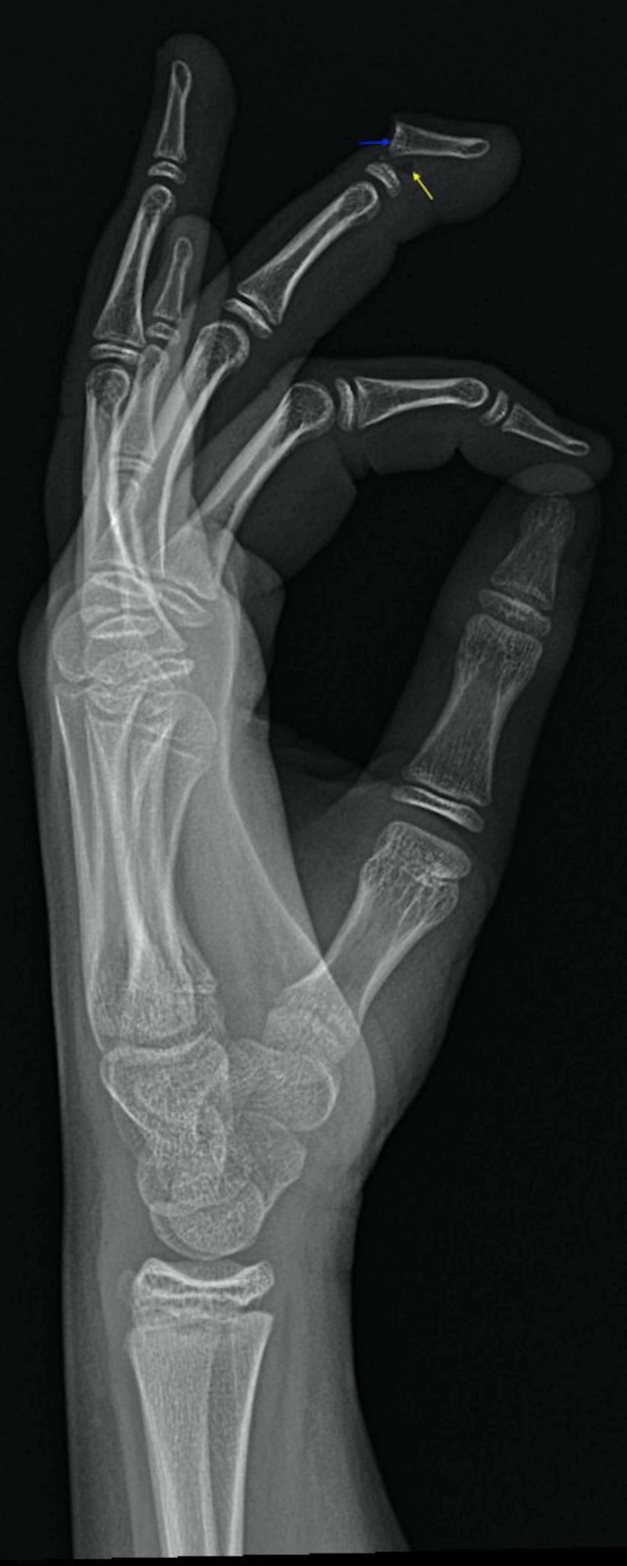
Radiograph marked transverse displacement of the distal phalanx (blue arrow), with small soft tissue gas noted at the fracture site (yellow arrow)

His vital signs included temperature of 98.1 °F, pulse of 98 beats per minute, respiratory rate of 16 breaths per minute, blood pressure of 117/73 mmHg, and oxygen saturation at 100% on room air. Physical examination revealed nail avulsion and ecchymosis. The sensation in the hand was intact. The radial pulse was also intact. The range of motion of the other fingers was also found to be intact. No laboratory studies were obtained. Radiographs demonstrated a severely displaced fracture through the epiphyseal plate of the base of the distal phalanx with a marked transverse displacement of the distal phalanx, with small soft tissue gas noted at the fracture site (Figure 2).

The wound was thoroughly irrigated with 250 ml of normal saline. No evidence of a foreign body was found. The patient’s tetanus status was up to date; he was given a weight-based dose of liquid ibuprofen for analgesia and one gram of cefazolin for the open fracture. The patient was transferred to the hand surgeon at the local children’s hospital due to the degree of displacement and the need for a thorough washout and closed reduction of the fracture.

## Discussion

A Seymour fracture can easily be overlooked as a minor injury to the nail. The nail bed laceration itself is usually not visible, but the proximal edge of the nail plate sits on top of the eponychial fold rather than beneath, making the nail appear “too long” in comparison to the other nails [1].

The pathophysiology, being similar to that of a mallet injury in adults, most commonly involves crushing injuries, sports injuries, and falls. This injury typically occurs when the distal phalanx of a fully extended digit undergoes forceful flexion, or when the distal phalanx experiences a crush injury [3]. Early evaluation and treatment have been the most effective in minimizing the risk of infection and any other complications [4]. It is important to consult orthopedics or a hand specialist, especially with pediatric patients. Open injuries may warrant operative intervention given that they are more prone to soft tissue infection and development of osteomyelitis [3]. Injury-related complications include osteomyelitis, distal phalanx growth disturbance, flexion deformity, and nail deformity [5]. Treatment-related complications like a secondary fracture displacement are uncommon but have been reported [6]. However, the majority of patients have good clinical outcomes.

Treatment for a Seymour fracture is dependent on whether it is an open or closed fracture. In the case of an open fracture that is prone to infection, it should be treated with operative irrigation, debridement, and intravenous antibiotics, along with a careful exploration to remove the proximal nail plate from the site of incarceration [5-6]. In closed fractures with severe soft tissue damage including the nail fold, the use of antibiotics is recommended [5]. Whenever possible, the nail should not be removed. The nail stabilizes the fracture, and the removal could make the injury more troublesome [6]. Interposed tissue should be removed from the fracture line. Late presentations of Seymour fracture often result in infection, growth arrest, and persistent mallet deformity of the distal phalanx [6].

## Conclusions

Seymour fractures are rare and often underestimated injuries that require early treatment. This case presentation highlights the common features of a Seymour fracture. The common pitfalls associated with this fracture are as follows: 1) clinicians tend to minimize workup in pediatric patients and 2) proximal nail injury can look deceptively benign.
